# Monitoring the Effects of Acupoint Antioxidant Intervention by Measuring Electrical Potential Difference along the Meridian

**DOI:** 10.1155/2015/286989

**Published:** 2015-03-15

**Authors:** Ming-Ming Xu, Jing-Ke Guo, Jin-Sen Xu, Chao-Xin Zhang, Shu-Tao Liu, Ri-Tao Liao, Chun-Tong Lin, Jian-Hui Guo, Ping-Fan Rao

**Affiliations:** ^1^CAS.SIBS-Zhejiang Gongshang University Joint Centre for Food and Nutrition Research, Zhejiang Gongshang University, Hangzhou 310018, China; ^2^Research Department of Meridian of Fujian Academy of TCM, Fuzhou 350003, China; ^3^College of Biological Science and Technology, Fuzhou University, Fuzhou 350108, China

## Abstract

Previous studies suggest that superoxide anions are possibly traveling along acupuncture meridians. The electrical potential difference (EPD) between acupoints may be related to the movement. To test the above hypothesis, we conducted a study investigating the effects of acupoint antioxidant interventions on the meridian EPD. Firstly, ST39 (L) and ST44 (L) were screened out for the EPD detection along the stomach meridian, and ST36 (L) was selected for interventions including acumassage with the control cream, as well as the TAT-SOD cream for 30 minutes, or injection with reduced glutathione sodium. The EPD between ST39 and ST44 was recorded for 80 minutes and measured again 48 h later. While the EPD increased during the acumassage, the acumassage with TAT-SOD cream and the glutathione injection generated waves of EPD increased, indicating the migration or removal from the visceral organ of a greater quantity of superoxide. Remarkably lower EPD readings 48 h later with both antioxidant acupoint interventions than the mere acumassage imply a more complete superoxide flushing out due to the restored superoxide pathway at the acupoint after interventions. The results confirm superoxide transportation along the meridians and demonstrate a possibility of acupoint EPD measurement as a tool to monitor changes in the meridians and acupoints.

## 1. Introduction

Reactive oxygen species (ROS), molecules or ions formed by the incomplete one-electron reduction of oxygen, have drawn considerable interest because of their regulations ranging from cell signaling to inflammatory response and cell death [1, 2]. An imbalance between ROS and cell defense systems results in the oxidative stress which is intricately connected to ageing and life span [3, 4]. While investigating the ROS distribution in living SD-rats in 2008, we accidentally discovered that there existed a special ROS-containing cellular network which was perfectly superimposable on a standard human acupuncture meridian network, as to the phenomenon that conception vessel meridian, spleen meridian, stomach meridian, and kidney meridian emitted intense green fluorescence corresponding to the intracellular ROS indicator [5]. It suggests that acupuncture meridian could be a channel in which ROS are either localized or transported.

Acupoints lie along the meridians. What acupoints will be in relation to this “ROS network”? In the issue of Free Radical Biology and Medicine, a controlled study [6] has shown that topical application of superoxide dismutase (SOD) fused with the TAT peptide (TAT-SOD), to various acupoints along the meridian lines used in acupuncture to treat obesity, leads to significant weight loss. Similarly, topical application of TAT-SOD on acupoint LI20 (Yingxiang) alleviated allergic rhinitis [7]. It indicates a possibility of the new method as a simple substitute to acupuncture and an insight of superoxide modulation along meridians for acupuncture mechanism. Therefore, it could possibly be assumed that acupoints would be the important sites that can store ROS, more specifically, superoxide as implied by the effect of TAT-SOD, and also be the gates through which ROS are transported. While the sites pile up with superoxide, “superoxide network” would be clogged with traffic. Thus, we can clear out the drains by way of the enzymatic removal of the intracellular superoxide at acupoints.

Within the acupuncture community, it is a commonly held opinion that acupuncture points have distinct electrical properties, for example, increased conductance [8, 9], reduced impedance and resistance [10, 11], and increased capacitance [8] compared to nonacupuncture points. Electrical measurements to study acupuncture points and meridians have become universal and internationally recognized. Recently, a clinical research [12] demonstrated that the EPD between ST39 and ST44 of essential hypertension group was between −60.00 mV and 60.00 mV, while the normal group was between −30.00 mV and 30.00 mV. The study revealed that the values of the EPD between acupoints can also reflect health status for subjects' organs.

Superoxide is negatively charged, and its migration is driven by the voltage difference. Therefore, any antioxidant intervention at acupoints is possible to be monitored by the electrical potential difference between acupoints along the meridians. To test the hypothesis, the effects of antioxidant interventions at one acupoint on the EPD between another two acupoints along the stomach meridian are investigated in hope to confirm superoxide's involvement in meridians and establish a new and effective tool for the studies of meridians and superoxide.

## 2. Materials and Methods

### 2.1. Materials

The vehicle cream for TAT-SOD cream was also used as control cream. The vehicle cream was baby lotion (Johnson & Johnson, Shanghai, China), which contains water, propylene glycol, myristyl myristate, glyceryl stearate, oleic acid, and stearic acid. 3000 U SOD/mL TAT-SOD cream was prepared by the homogenization of membrane permeable TAT-SOD with the vehicle cream. TAT-SOD was prepared by recombinant expression of a fusion protein of human Cu, Zn-SOD fused with TAT peptide in* E. coli* as follows: constructs preparation: the nucleic acid sequence encoding TAT-SOD fusion protein was constructed by DNA recombinant technology and inserted into expression vector pGEX-2 T; cell culture and transfections:* E. coli* (BL21, DH5*α*) cells were transformed with the expression vector pGEX-2 T containing the inserted TAT-SOD; TAT-SOD fusion protein preparation: TAT-SOD was expressed in the* E. coli* by the induction of IPTG. After purification of affinity chromatography, electrophoretically pure TAT-SOD protein was obtained [13]. 0.12 g/mL reduced glutathione sodium (Laboratorio Farmaceutico C.T.S.R.L., Strada Solaro, Villa Sayonara, Sanremo, Italy) was prepared by injecting 5 mL 0.9% (w/v) saline into a germ-free bottle equipped with 0.6 g freeze-dried powder of reduced glutathione sodium. Disposable sterilized acupuncture needles of 300 *μ*m diameter and 40 mm length stainless steel (Huacheng, China) were used for acupuncture.

### 2.2. Subject

A total of 30 healthy volunteers were recruited in this study after giving full informed consent. Participants should meet the following criteria: (1) 20 and 35 years of age; (2) no history or physical examination suggestive of renal, hepatic, or cardiovascular diseases or any other severe organic diseases; (3) having regular diet; minimal liquor, tobacco, tea, and coffee; normal sleeping patterns (before 12 a.m.); (4) no long-term medications; (5) no history of drug abuse; (6) having not undergone acupuncture or other acupoint interventions within 1 month before the test. This study was approved by the Medical Research Ethics Committee and Institutional Review Board of Fujian Institute of Traditional Chinese Medicine.

### 2.3. Experimental Protocol

Before the trial, several acupoints along the ST meridian were selected for their convenience to operate. The subjects first underwent the measurement of EPD between acupoints. Based on the stability and individual difference of the EPD between acupoints, two acupoints were screened out for the EPD detection along the ST meridian. Meanwhile, one downstream acupoint along the ST meridian for interventions was selected. Subsequently, 30 subjects were divided into 6 groups. The treatments applied to different groups on acupoint were as follows: (A) no treatment, (B) acumassage without cream, (C) acumassage with control cream, (D) acumassage with TAT-SOD cream (3000 U/mL), (E) injection of reduced glutathione sodium injection (100 *μ*L, 0.12 g/mL), and (F) acupuncture. When the EPD between the two selected detection acupoints was relatively stable after a balance of about 20 min, the cream was applied on the intervention acupoint assisted by massage stick for 30 min, reduced glutathione sodium was injected into the intervention acupoint quickly, and acupuncture was conducted, respectively. Make a record of the EPD between the two detection acupoints for 60 min, since the start-time of acupoint intervention. Besides, the EPD between the same acupoints was monitored again 48 h after treatments.

### 2.4. Selection of EPD Detection Points of Stomach Meridian

Stomach meridian has 45 acupoints each side. Ruling out the acupoints in the face, head, torso, and fingertip that are inconvenient to operate, 5 acupoints as EPD detection acupoints were selected (Table 1).

### 2.5. EPD Monitoring Procedure

The subjects were conscious, placed in a supine position, and asked to breathe calmly. The acupoints were localized according to name and location of acupoints: Chinese National Standards GB/T12346 [14]. The hair on the selected detection acupoints was trimmed. After previously disinfected with medical alcohol, the point sites were connected to a digital potentiometer via Ag/AgCl disposable ECG electrodes. After 15–25 min of equilibrium, data of the EPD between two acupoints in 10 min was collected and recorded for selection of two acupoints for the EPD detection along the ST meridian. During the monitoring of acupoint intervention, the EPD was constantly recorded for about 80 min.

### 2.6. Acumassage Methods

Massage stick (YJ-8, Bailing, China) was used for acupoint massage. Acupoint for intervention was localized according to name and location of acupoints: Chinese National Standards GB/T12346 [14]. B group was immediately massaged assisted by massage stick. C and D groups have first applied 0.2 mL of the control cream and TAT-SOD cream, respectively, in an area of 1 cm^2^ to acupoint for intervention and then massaged assisted by massage stick in a minute. During the 30 min, physician repeated this manipulation for 3 min every 5 min.

### 2.7. Acupuncture Methods

Disposable sterilized acupuncture needles of 300 *μ*m diameter and 40 mm length stainless steel (Huacheng, China) were used for acupuncture in this study. Acupoint for intervention was localized according to name and location of acupoints: Chinese National Standards GB/T12346 [14]. Skin was disinfected with medical alcohol. The acupuncture procedures were referred to in Chunxiao Wu's paper [15]. After manipulating the needle for 10 min, the needle was held in place for another 20 min. After that, the needle was removed quickly.

### 2.8. Statistical Analysis

Data are reported as means (SEM). All statistical analyses were carried out using SPSS version 21.0 software. Results in left EPD and right EPD of the two same detection acupoints were compared using two-sample *t*-test, and paired *t*-test was used to analyze interclass variance. A one-factor ANOVA (SPSS version 21.0), followed by Duncan's test, was used to test for significant differences of the reduction of the EPD of each group 48 h after treatments.

## 3. Results

### 3.1. Selection of Two Detection Points for the EPD of Stomach Meridian and One Point for Interventions

As shown in Table 2, the stability of the EPD between acupoints varied in different combinations. Of all detection points, the EPD between ST37 and ST42 (R) and that between ST42 and ST44 (L) fluctuated relatively larger, and the EPD between ST39 and ST44 (L) was most stable. As to *T*_1_ test (paired *t*-test), the EPD of most acupoint combinations showed significant individual difference; only the EPD between ST39 and ST44 (L) showed no significances (*P* > 0.05). There are also generally existing differences between the left side and the right side of the same two acupoints, regarding *T*_2_ test (two-sample *t*-test).

Therefore, ST39 and ST44 (L) were screened out for the EPD detection along the ST meridian for the stable and reliable voltage reading. In the meantime, the downstream acupoint ST36, admittedly an important and effective acupoint along the ST meridian in dredging meridian channels and relieving fatigue [16–18], was selected for interventions.

### 3.2. The Effect of Acupoint Intervention on the EPD between Acupoints

**Figure 1:**
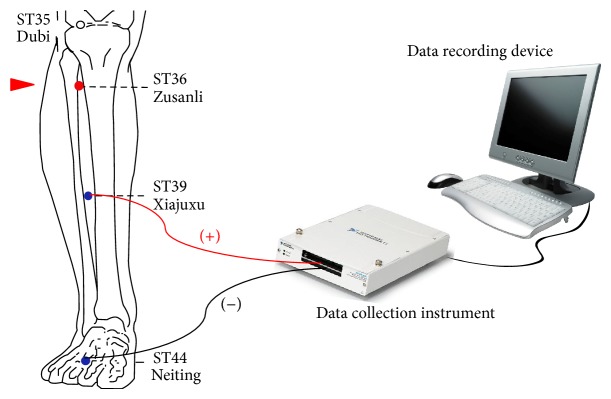
A diagrammatic sketch of how to monitor the variation of the EPD between ST39 and ST44 caused by different acupoint interventions on ST36.

A diagrammatic sketch of how to monitor the variation of the EPD between ST39 and ST44 caused by different acupoint interventions on ST36 was shown in Figure 1.

**Figure 2:**
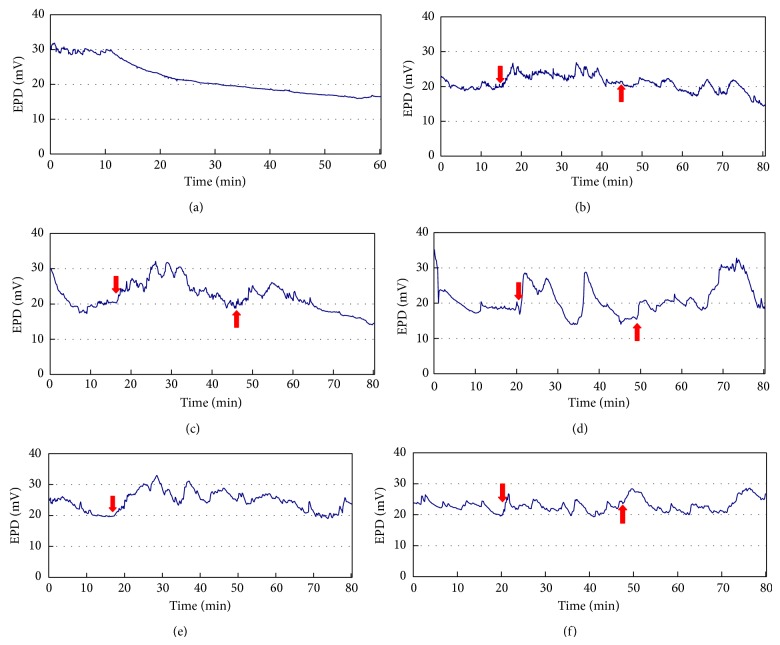
Monitoring the EPD caused by acupoint antioxidant interventions and acupuncture. (a) group A: no treatment; (b) group B: acumassage without cream; (c) group C: acumassage with control cream; (d) group D: acumassage with TAT-SOD cream (3000 U/mL); (e) group E: injection of reduced glutathione sodium (100 *μ*L, 0.12 g/mL); and (f) group F: acupuncture. Downward arrow: start-time of intervention; upward arrow: end-time of intervention.

ST36 was subject to acumassage alone, acumassage with the control cream, and the cream containing membrane permeable TAT-superoxide dismutase for 30 min or injected with reduced glutathione sodium (100 *μ*L, 0.12 g/mL), and the EPD between ST39 and ST44 was recorded for 80 min and measured again 48 h later. The results of the variation trends of different interventions during the first 80 min were presented in Figure 2.

After 15–25 min of equilibrium, the EPD of the control group, group A, displayed a slight decrease in the next 60 min. Groups other than A group showed a significant increase in EPD reading right after ST36 was stimulated with different modes. These groups all fluctuated during a 30-min period of intervention. However, the EPD readings of B and C groups gradually descended when interventions ended, while D, E, and F groups still fluctuated at high levels in the next 30 min follow-up. The variation trend of B and C groups was similar.

The reduction of EPD between ST39 and ST44 of each group 48 h after the intervention was demonstrated in Table 3. Compared to A group of no treatment on acupoint, B group of acumassage without cream and C group of acumassage with control cream showed almost no decrease, while D group of acumassage with TAT-SOD cream, E group of reduced glutathione sodium injection on acupoint, and F group of acupuncture reported a very significant decline (*P* < 0.01). The fall of the EPD readings of D and F groups was similar without significance (*P* < 0.01). E group had the largest decline between all groups (*P* < 0.01).

## 4. Discussion

TAT-SOD, a fusion protein of human Cu, Zn-SOD fused with TAT peptide, is permeable membrane and well capable of eliminating intracellular superoxide. As a negatively charged anion, superoxide migration is caused by the EPD. Therefore, the EPD between acupoints may reflect the migration of superoxide along the meridian. The phenomenon that the EPD readings of B and C groups gradually descended when interventions ended, while D and E groups still fluctuated at high levels in the next 30 min follow-up, indicates that ROS, or more specifically superoxide anion, may be traveling along meridians.

All the interventions at ST36 in this study resulted in the immediate increase in EPD reading. The elevation of EPD reading stopped almost right after the interventions stopped with the acumassage alone and the acumassage with the control lotion, indicating that the intervention at ST36 could cause the increased flow of superoxide anion along the meridians. With both antioxidant oxidant intervention and acupuncture, it is clear that the increased superoxide flow could last for some time even after the intervention stopped. The continuation of the elevated flow of superoxide resulted in a lower EPD reading 48 h later, implying a lower level of superoxide migration as the result of the intervention. ST36 is like a gating point modulating the superoxide flow from its source organ. It turned on while acumassage intervention lasted but went off as soon as it stopped. Antioxidant interventions and acupuncture seemed to generate a lasting effect to turn on the connection so as to enable the flow of waves of superoxide even after the intervention stopped, so much as to clear up the storage of superoxide at its source, as indicated by the remarkable lower EPD level 48 h later.

**Figure 3:**
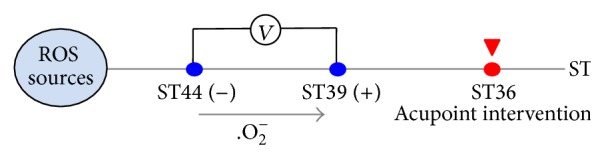
Schematics of a possible involvement of the acupoint superoxide removal.

Figure 3 depicts schematics of a possible involvement of the acupoint superoxide removal. The positive electrode and the negative electrode were attached to ST39 and ST44, respectively. The EPD readings between ST39 and ST44 were positive, which meant that superoxide migrated from ST44 to ST39. When ST36 was subject to acupoint intervention, the EPD readings increased rapidly and then fluctuated at high levels; that is to say, more superoxide from upstream ROS sources migrated from ST44 to ST39 when a portion of superoxide piled up at ST36 was cleared out or flowed out by acupoint intervention, for example, mere acumassage, acumassage with control cream, or acumassage with TAT-SOD cream.

In addition, the EPD readings were much lower with both antioxidant acupoint interventions than the mere acumassage 48 h later, implying a more potent superoxide flushing out due to the restored superoxide pathway at the acupoint after the interventions. The effect of acumassage with TAT-SOD cream was similar to that of acupuncture intervention, as shown in Figure 3 and Table 2. The acupuncture at ST36 resulted in similar EPD patterns and 48 h EPD drop with the antioxidant interventions, suggesting a possible involvement of the acupoint superoxide removal.

Moreover, acupoint EPD measurement can quickly respond synchronously, when the status of intervention changes. For example, after acupuncture intervention at ST36, the needle was removed quickly; in the meantime, the EPD reading between ST39 and ST44 sharply ascended. There the same thing happened at the start-time of interventions. Therefore, acupoint EPD measurement hold great potential as a powerful tool to monitor changes in the meridian and acupoints.

Different responses of superoxide flow along the meridian caused by different interventions may be attributed to the biological nature of acupoints. It is reported that acupoints are high in mast cells which will respond to pressures such as acumassage [19]. Meridians are considered to anatomically reside in the fascia, connective tissues containing fibroblasts [20]. Fibroblasts in fascia are known to convert from network status with high conductivity to disconnected globular status under ROS stimulation. It is possible that the antioxidant interventions at acupoints lower than the intracellular superoxide level and restore the fibroblasts to the network status to recover the connectivity of superoxide flow. Much work is necessary to verify the reasoning and to reveal cellular mechanism of antioxidant interventions.

## 5. Conclusions

The results confirm superoxide transportation along the meridian and demonstrate a great potential of acupoint EPD measurement as a powerful tool to monitor changes in the meridians and acupoints.

## Figures and Tables

**Table 1:** EPD detection acupoints of ST and their anatomical positions.

Acupoints	Location
ST36: Zusanli	On the anterior lateral side of the leg, 3 Cun below ST35 Dubi^*^, one finger breadth (middle finger) from the anterior crest of the tibia
ST37: Shangjuxu	On the anterolateral side of the leg, 6 Cun below ST35 Dubi, one finger breadth (middle finger) from the anterior crest of the tibia
ST39: Xiajuxu	On the anterolateral side of the leg, 9 Cun below ST35 Dubi, one finger breadth (middle finger) from the anterior crest of the tibia
ST42: Chongyang	On the dome of the instep of the foot, between the tendons of the long extensor muscle of the big toe and the long extensor muscle of the toes, where the pulsation of the dorsal artery of the foot is palpable
ST44: Neiting	On the instep of the foot, in the depression distal to the commissure of the 2nd and 3rd metatarsal bones

^*^Location of ST35 Dubi: with the knee flexed, on the knee, in the depression lateral to the patella and its ligament.

**Table 2:** The interclass variance and between-cluster variance of the EPD of acupoints along the ST meridian.

Acupoints	Interclass variance	Between-cluster variance	*T*_1_	*T*_2_
ST36-ST37 (L)	2.07	6.48	∗∗	∗
ST36-ST37 (R)	1.31	7.19	∗∗	
ST36–ST39 (L)	1.02	8.31	∗∗	∗∗
ST36–ST39 (R)	1.38	7.22	∗∗	
ST36–ST42 (L)	2.54	5.68	∗∗	∗∗
ST36–ST42 (R)	1.26	6.28	∗∗	
ST36–ST44 (L)	1.33	7.10	∗∗	∗∗
ST36–ST44 (R)	1.79	4.31	∗∗	
ST37–ST39 (L)	1.91	7.79	∗∗	∗∗
ST37–ST39 (R)	1.46	7.71	∗∗	
ST37–ST42 (L)	1.92	8.18	∗∗	∗∗
ST37–ST42 (R)	3.90	6.16	∗∗	
ST37–ST44 (L)	1.29	6.27	∗∗	∗∗
ST37–ST44 (R)	1.14	6.18	∗∗	
ST39–ST42 (L)	2.30	7.20	∗∗	∗
ST39–ST42 (R)	1.29	7.46	∗∗	
**ST39**–**ST44 (L)**	**1.01**	**1.08**		∗∗
ST39–ST44 (R)	2.15	5.41	∗∗	
ST42–ST44 (L)	3.18	6.21	∗∗	∗∗
ST42–ST44 (R)	2.48	5.20	∗∗	

*T*_1_ (paired *t*-test): comparing the EPD of the same points of different subjects.

*T*_2_ (two-sample *t*-test): comparing the left EPD and right EPD of the same detection points.

^**^(*P* < 0.01): regarded as very significant.

^*^(*P* < 0.05): regarded as significant.

**Table 3:** The reduction of EPD 48 h after treatments.

Treatment		The reduction of EPD (mV)
A	No treatment on acupoint	0.34 ± 1.67a^#^
B	Acumassage without cream	1.05 ± 1.96a
C	Acumassage with control cream	1.00 ± 1.75a
D	Acumassage with TAT-SOD cream	4.51 ± 0.51b
E	Reduced glutathione sodium injection on acupoint	7.79 ± 0.64c
F	Acupuncture	4.35 ± 0.38b

^#^Values in the same column with different letters were significantly different by Duncan's test (*P* < 0.01).
